# Redefining the boundary between crystalline and sedimentary rock of Eastern Dahomey Basin

**DOI:** 10.1038/s41598-021-84687-8

**Published:** 2021-03-03

**Authors:** Ganiyu O. Mosuro, Niyi-Ola Adebisi, Stephen O. Ariyo, Kamaldeen O. Omosanya, Olateju O. Bayewu, Moroof O. Oloruntola

**Affiliations:** 1 Department of Earth Sciences, Olabisi Onabanjo University, Ago-Iwoye, Nigeria; 2 Oasisgeokonsult, 7052 Trondheim, Norway; 3 Department of Geosciences, University of Lagos, Lagos, Nigeria

**Keywords:** Environmental sciences, Solid Earth sciences

## Abstract

This study defines a new boundary between the crystalline and sedimentary rocks of Eastern Dahomey Basin at the southwestern part of Nigeria using a geophysical approach that combines regional aeromagnetic and ground resistivity data. Aeromagnetic data covering the entire Eastern Dahomey Basin were acquired at 500 m line spacing, 80 m mean terrain, and processed into grids of Residual Magnetic Intensity (RMI) map. Filters and corrections such as upward continuation, and reduction to equator were applied to enhance deep magnetic sources and correct for magnetic inclination and declination. Tilt Derivative Angles (TDR) was applied for edge detection. To support the aeromagnetic analysis and interpretation, 104 Vertical Electrical Sounding (VES) surveys and 8 Electrical Resistivity Tomography (ERT) data were also acquired, processed and interpreted along the basement-sedimentary rock boundary. The TDR revealed a significant trend that corresponds to the edge between the basement complex and the sediments of the Eastern Dahomey Basin. A strong match was also noticed between the VES positions and the TDR map. Areas interpreted as basement rocks from the VES stations align with positive values on the TDR maps while the sedimentary terrains have negative TDR values. Our work demonstrates that areas that were previously fixed as sedimentary terrains on geological maps belong to the crystalline basement or transition zone. A new and reliable geological boundary is hereby drawn between the basement and sedimentary rocks. Thus, providing a revised map of the Eastern Dahomey Basin.

## Introduction

Geological maps have been described by^1^ as classical forms of spatial information delineating different stratigraphic or rock units. As geological maps are frequently made from regional studies, field observations, interpretation of borehole records, and remotely sensed data^1,2^, their accuracy is often dependent on the abundance of outcrop exposures. Borehole data provides one dimensional (1-D) information and are only representative of a single point down the earth surface while the interpretation of remotely sensed data is subjective in nature. Hence, geological boundaries are fixed based on limited information^3–6^. To correct for errors in geological maps, an integrated approach that incorporates geologic, geophysical, remote sensing, and GIS techniques is recommended^7^.

Potential field methods such as aeromagnetic and resistivity methods^8–13^ offers complementary data to conventional field-based geological mapping techniques when making geologic maps. They can provide continuous coverage of an entire area, detailed analysis of subsurface structures and stratigraphy^12^. Aeromagnetic signatures are highly dependent on the underlying rock types above which measurement are made^14–16^ and with resistivity methods can yield information on differences in the earth’s rock electrical and magnetic properties. Considering that sedimentary rocks have lower magnetic susceptibility than crystalline rocks, with intermediate and basic igneous rocks having higher magnetic susceptibility than acidic igneous rocks^17^. Resistivity of stratified rocks are also in several orders higher than those of crystalline rocks^18–21^.

**Figure 1:**
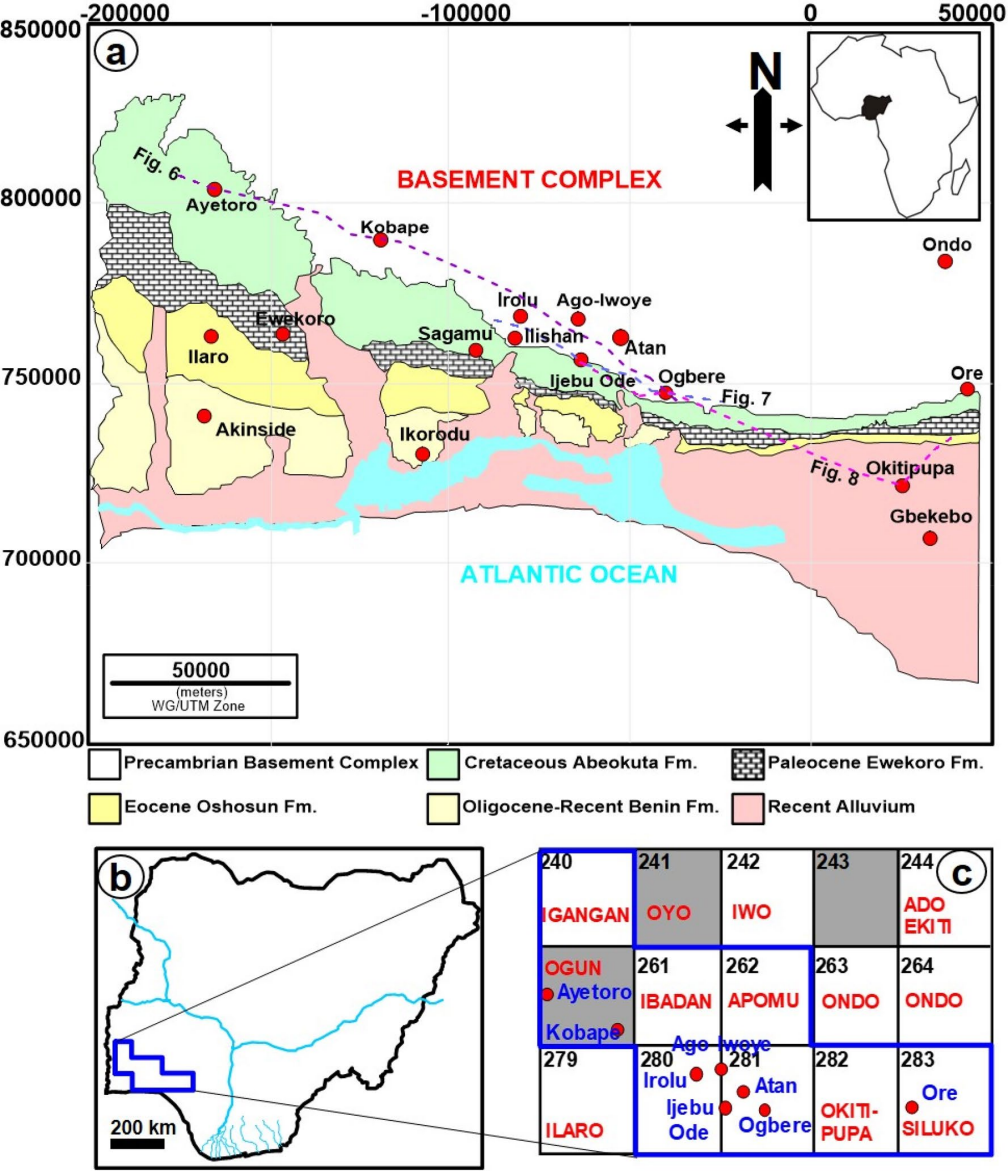
(**a**) Geologic and location map of the study area showing the major localities discussed in the text and also the VES section lines in Figs. 6, 7 and 8. Inset map shows location of Nigeria within the context of Africa*.* (**b**) Map of Nigeria showing the location of the aeromagnetic grids (blue polygon) used in this study. (**c**) These are 240-Igangan, 260-Ogun, 261-Ibadan, 280-Lagos, 262-Apomu, 281-Lekki, 282-Okitipupa, and 283-Siluko. Note: (**a**) and (**b**) were drawn in by CorelDRAW2016, https://www.corel.com/en/

The Eastern Dahomey Basin covers Benin and western Nigeria (Fig. 1) and was formed during the opening of the Equatorial Atlantic and separation of Laurasia and Gondwana in Late Jurassic to Cretaceous^22–24^. The most recent map of the basin showing its boundary from the northern bound crystalline basement complex and easternmost part in Nigeria is credited to^25^. However, recent geophysical surveys in several localities such as Kobape, Ijesha-Ijebu-Irolu, Atan-Ijebu-Ode and Ogbere (this work) revealed that the boundary between the basement complex-sedimentary rocks (BS) was subjectively fixed by previous authors such as^25^. Perhaps, earlier geologic mappings in the area may be limited by the sparse availability of borehole data and outcrop exposure in the western part of Nigeria as compared to the remainder of the Dahomey Basin in Togo, Ghana and Benin^22,23^. This study therefore provides a new definition of the transition and boundary between the crystalline-sedimentary rocks of the Eastern Dahomey Basin by incorporating aeromagnetic data with ground electrical resistivity survey. The interpretation results from magnetics and resistivity presented in this paper supplement existing geologic data and knowledge in the area, with wider implications for understanding the evolution of the Eastern Dahomey Basin.

## Brief history of the Dahomey Basin

### Geologic setting

**Figure 2:**
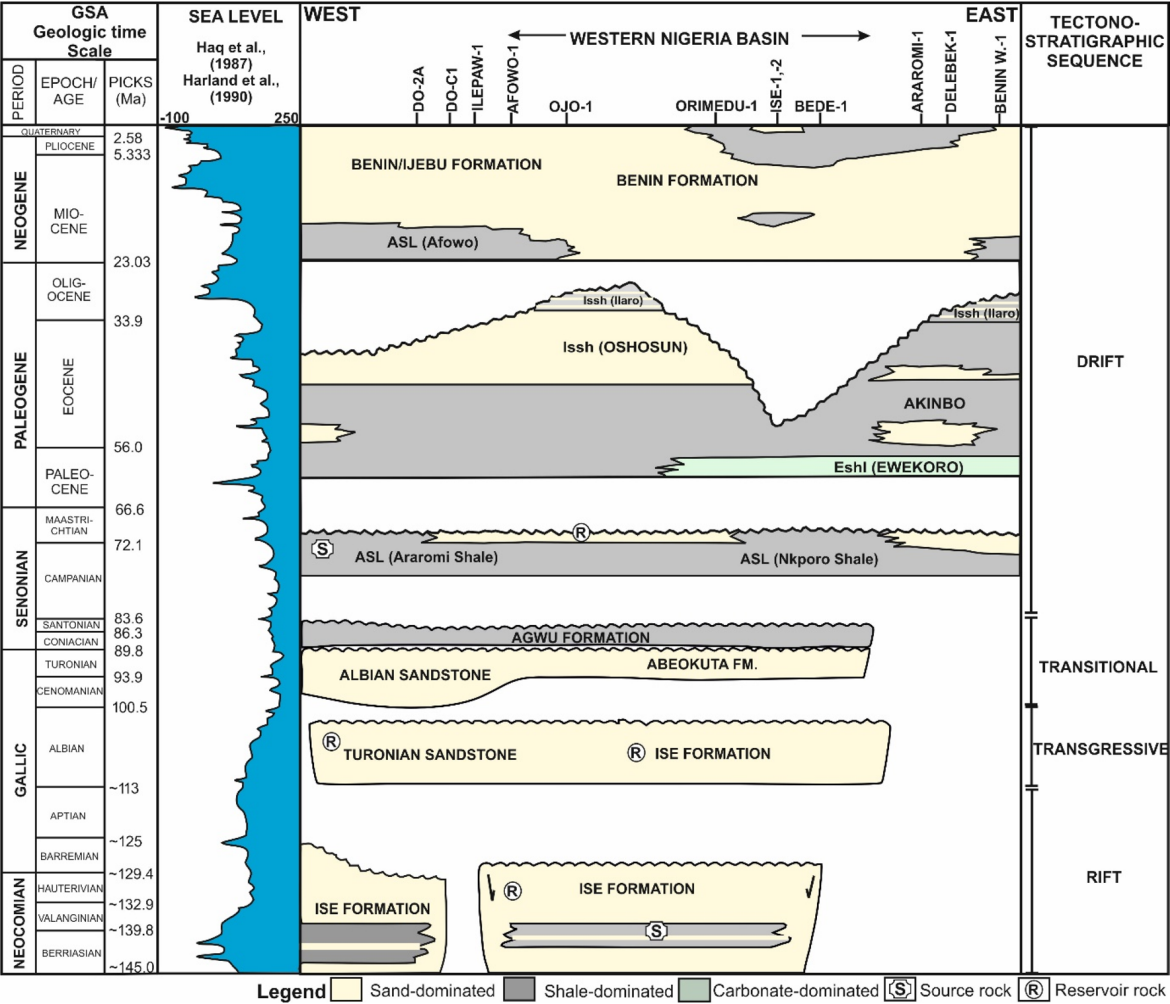
Chrono-stratigraphic column of the Eastern Dahomey Basin modified from^27,71^. The stratigraphic infill of the Dahomey Basin varies dramatically from both onshore and offshore areas^24^ and reflects four main tectonic stages i.e., pre-rift (Late Jurassic), rift (Neocomian-Lower Cretaceous), transitional (Cenomanian-Santonian) and post-rift (Maastrichtian-Holocene). The figure also highlights the elements of the petroleum system of the basin from the west (Benin) to the east (western Nigeria) as revealed by wellbores DO-2A, DO-C1, Ilepaw-1, Afowo-1, Ojo-1, Orimedu-1, Ise-1,-2, Bede-1, Araromi-1, Delebek-1, and Benin W-1^32^. The sea level curve is after^94,95^.

The study area is located within the Southwestern part of Nigeria and extends for about 300 km from Ayetoro through Ishara, Ijebu ode, Ijebu Ife, Ogbere to Okitipupa, the easternmost part of the Eastern Dahomey Basin (Fig. 1a). Geologically, the study area is part of the Dahomey embayment, which extends from the Volta region of Accra, Ghana through Togo and Benin Republic to the Southwestern part of Nigeria where it terminates against the Okitipupa ridge^26–28^. The Eastern Dahomey Basin is bounded by the Basement Complex in the north and in the south by the Atlantic Ocean (Fig. 1a). The Dahomey embayment is a marginal basin formed during the separation of the African and South America plate in Early Cretaceous, around 125 Ma/magnetic anomaly M0^24,29–31^. Although, the stratigraphic infill of the Dahomey Basin varies dramatically from both onshore and offshore areas^22^, the overall structural evolution of the basin is complicated and aligned along four main sedimentary sequences namely pre-rift (Late Jurassic), rift (Neocomian-Lower Cretaceous), transitional (Cenomanian-Santonian) and post-rift (Maastrichtian-Holocene) sequences (Fig. 2). These four tectono-sedimentary packages correspond to intracratonic, rift, transitional and drift stages^23^. The drift stage was preceded by a Late Cretaceous transitional period which extends from Cenomanian to Santonian^22^ (Fig. 2).

### Stratigraphic succession of the Dahomey Basin

The lithostratigraphic succession of the Dahomey Basin are documented and known from wellbores drilled in Benin Republic and in western Nigeria, offshore basins (Fig. 2). Sedimentary infill of the basin include terrestrial sediments, shallow marine, deep marine to open deep marine, and shelf sediments that are prograding southwards from bottom to top^32^. The oldest succession is the Cretaceous or Abeokuta Group (Neocomian to Albian) drilled in the Ise-1 and Ise-2 wellbores^33^ and in offshore Benin Republic^22^. The Abeokuta Group overlies the basement rocks in the entire basin and is made up of a sequence of folded conglomerates that are overlain by sandstones and sands with kaolinitic clays intercalation^34^. The Abeokuta Group is further divided into the Ise Formation (Early Cretaceous rift sequence), the Afowo or the Agwu Formations, comprising of Cenomanian shales and Turonian sandstone” unit^22^, and the Campanian to Maastrichtian Araromi Formation, which has its time equivalent as the ‘Npkoro Shale’ in the Anambra Basin (Fig. 2). Above the Abeokuta Group is the Paleocene-Lower Eocene Ewekoro Formation and the Eocene Akinbo Formation. The Akinbo Formation consists of shallow marine shale and clayey sequence^35^ while the Ewekoro Formation consists of coquinoidal limestone. The Imo Formation is equivalent to the Ewekoro Formation in the Eastern Dahomey Basin and also corresponds to the Akata Formation in the Niger Delta^34^. The Eocene Oshosun Formation overlies the Imo Formation and includes marine shales and sandy shales that is often dominated by phosphatic materials^22^. The Ilaro Formation on top of the Oshosun Formation consists of massive, fine-coarse, cross-bedded sandstones, clays and shales, occasionally with phosphate beds at its type locality in Ilaro (Fig. 2). The youngest stratigraphic unit of the entire Dahomey Basin from west to east is the Miocene to Recent Benin Formation, which comprises mainly of marine shelf sands containing shale sequences (Fig. 2).

## Results and interpretations

### Aeromagnetic data

**Figure 3:**
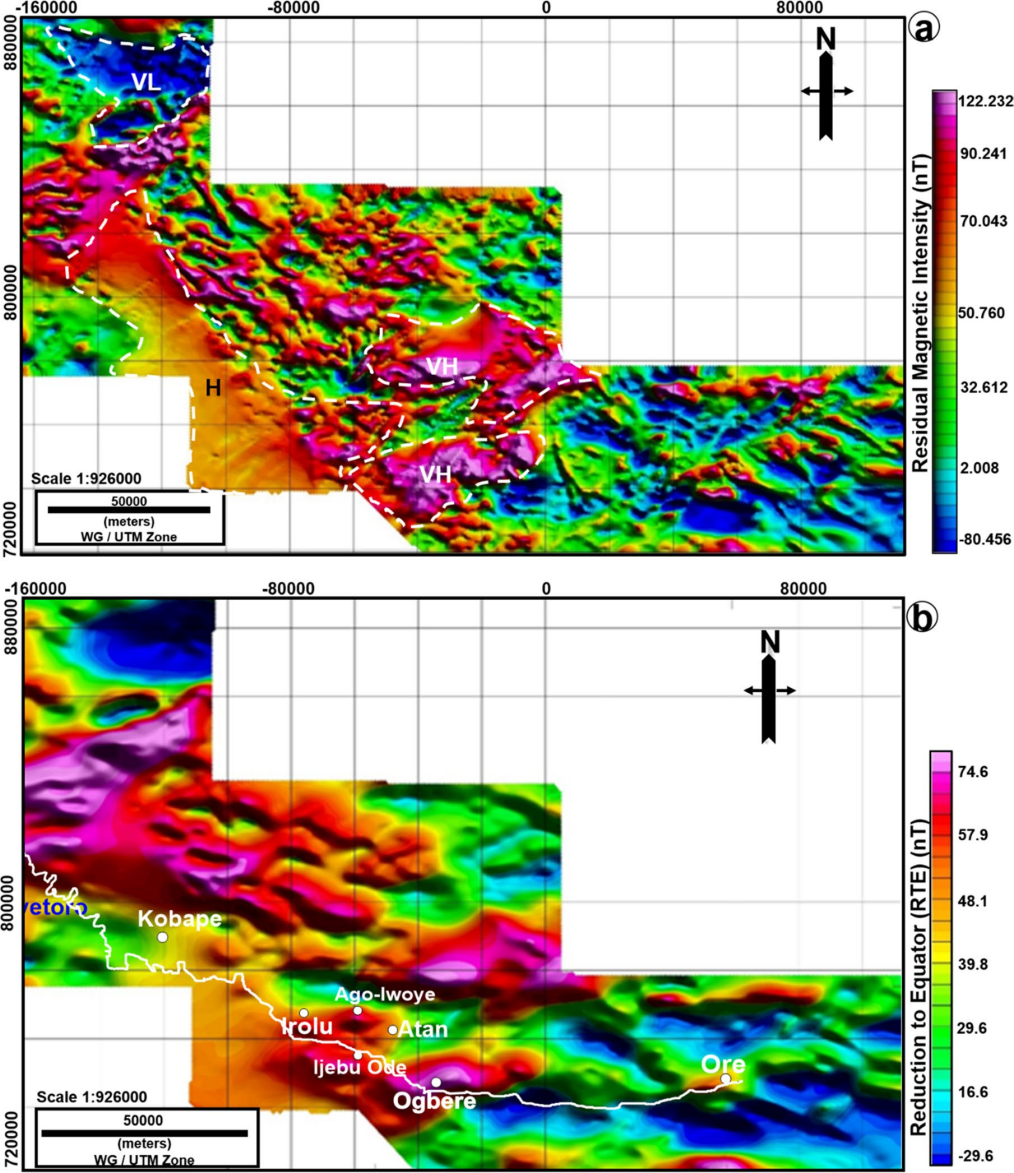
(**a**) Residual Magnetic Intensity (RMI) in nanoTesla (nT) grid displayed as a shaded relief map. Also shown in the map are the main localities for the VES and ERT surveys. The white line is the eastern limit of the sedimentary rocks of the Eastern Dahomey as defined by^25^. VL-very low, VH-very high and H-high anomalies. (**b**) Combined filtering using Reduction to Equator (RTE) to center the anomaly source and the Upward Continuation (UC) to enhance deep anomaly source in nT. The white line is the eastern limit of the Dahomey Basin as defined by^25^. The figure was made using Oasis Montaj software (https://www.seequent.com/products-solutions/geosoft-oasis-montaj/) and edited in CorelDRAW2017, https://www.corel.com/en/.

The Residual Magnetic Intensity (RMI), revealed areas with very high, high and very low magnetic gradients (Fig. 3a). The overall magnetic gradients on the RMI map are observed to vary from + 122 to − 80 nT (Fig. 3a). The very relatively low magnetic gradients are seen in areas intercepted between 0–100,000 m in the Easting direction and (720,000–780,000 m) in the Northing direction (Fig. 3a). The same low gradient trend is observed at the upper left of the gridded RMI at (− 160,000 to − 110,000 m) easting direction and (850,000 to 880,000) Northing direction (Fig. 3a). The highest values of magnetic gradient anomalies are observed in two main areas given by (− 60,000 to 0 m) Easting direction, (730,000 to 780,000 m) Northing direction and at (− 60,000 to 0) Easting-direction, (810,000 to 860,000) Northing -direction (Fig. 3a). These anomalies are interpreted as evidence for a magnetic basement.

By combining the transformed data with the geological boundary of ^25^ separating the basin from the crystalline basement complex (Fig. 3b), very high magnetic anomalies (58–75 nT) were observed in both the basement complex terrain and sedimentary basin (Fig. 3b). This further support the interpretation that the RMI anomalies are related to the magnetic basement. Although, there was no marked trend that could discriminate the basement rocks from the sedimentary rocks. An obvious decrease in magnetic gradient around the middle left and the center of the grid is likely associated with southwestern gradation from the basement into the sedimentary terrain (Fig. 3b). Based on visual inspection only, this would be the trend that separates the basement complex from the basin. However, the boundary line of the two rocks as drawn by^25^ is different when the geological map was overlain on the RMI. The Billman’s boundary (1976) transects areas with both relatively high and medium, and then into areas of low magnetic gradients (Fig. 3a). It is possible that this signifies that the RMI only measures the magnetic basement, which is not necessarily the same as the geological basement. The magnetic basement is the depth of the magnetic source, which is an estimate of the thickness of the overlying sediments^37^. An alternative interpretation is that the rock boundary of^25^ is simply inappropriate in some places.

**Figure 4:**
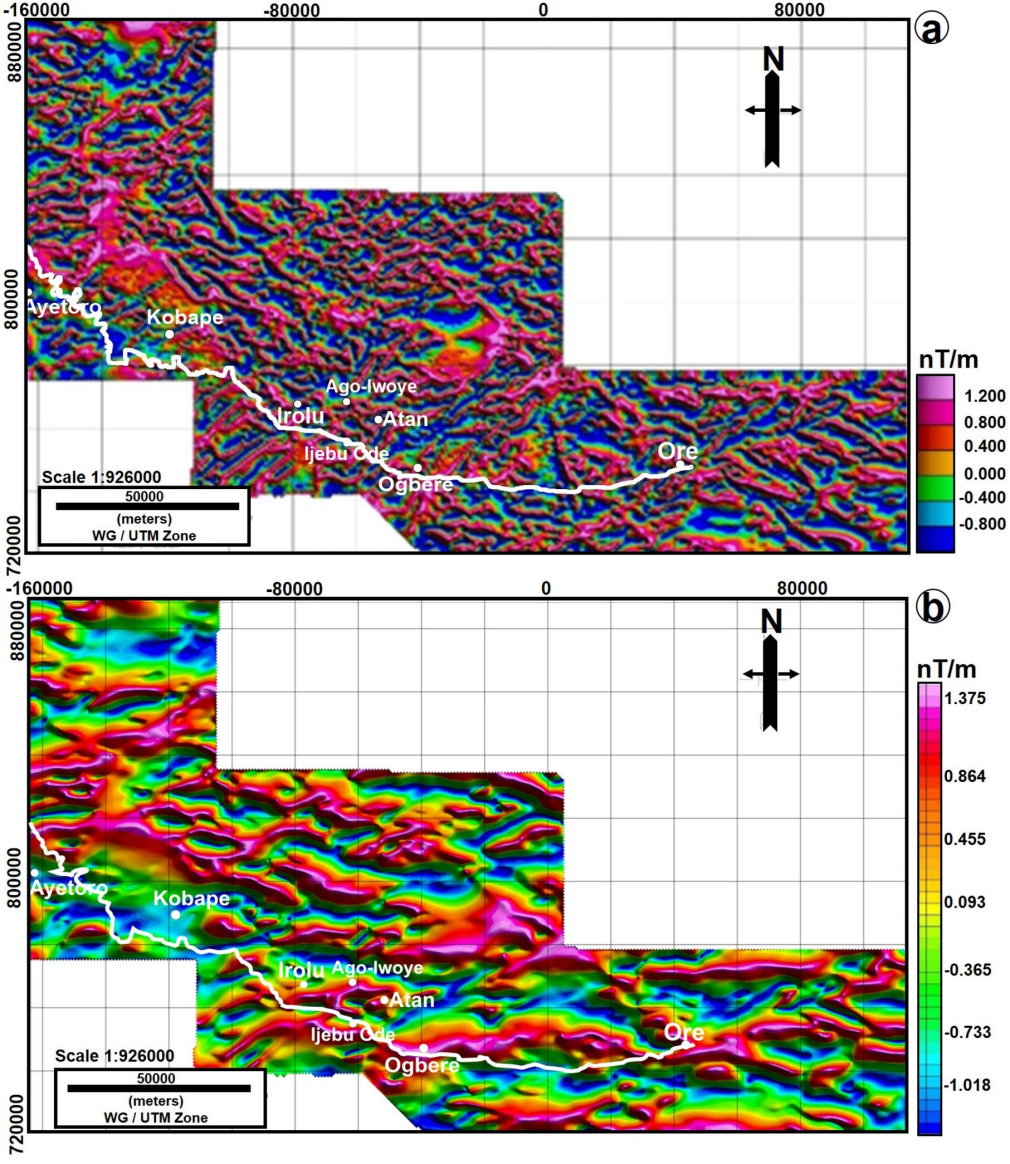
(**a**) Tilt Derivatives of the RMI show similar trend as that observed on the RMI. This is at variance with the geologic boundary of^36^. (**b**) Tilt Derivatives of the RTE and Upward Continuation of 2000 m. The figure was made using Oasis Montaj software (https://www.seequent.com/products-solutions/geosoft-oasis-montaj/) and edited in CorelDRAW2017, https://www.corel.com/en/.

**Figure 5:**
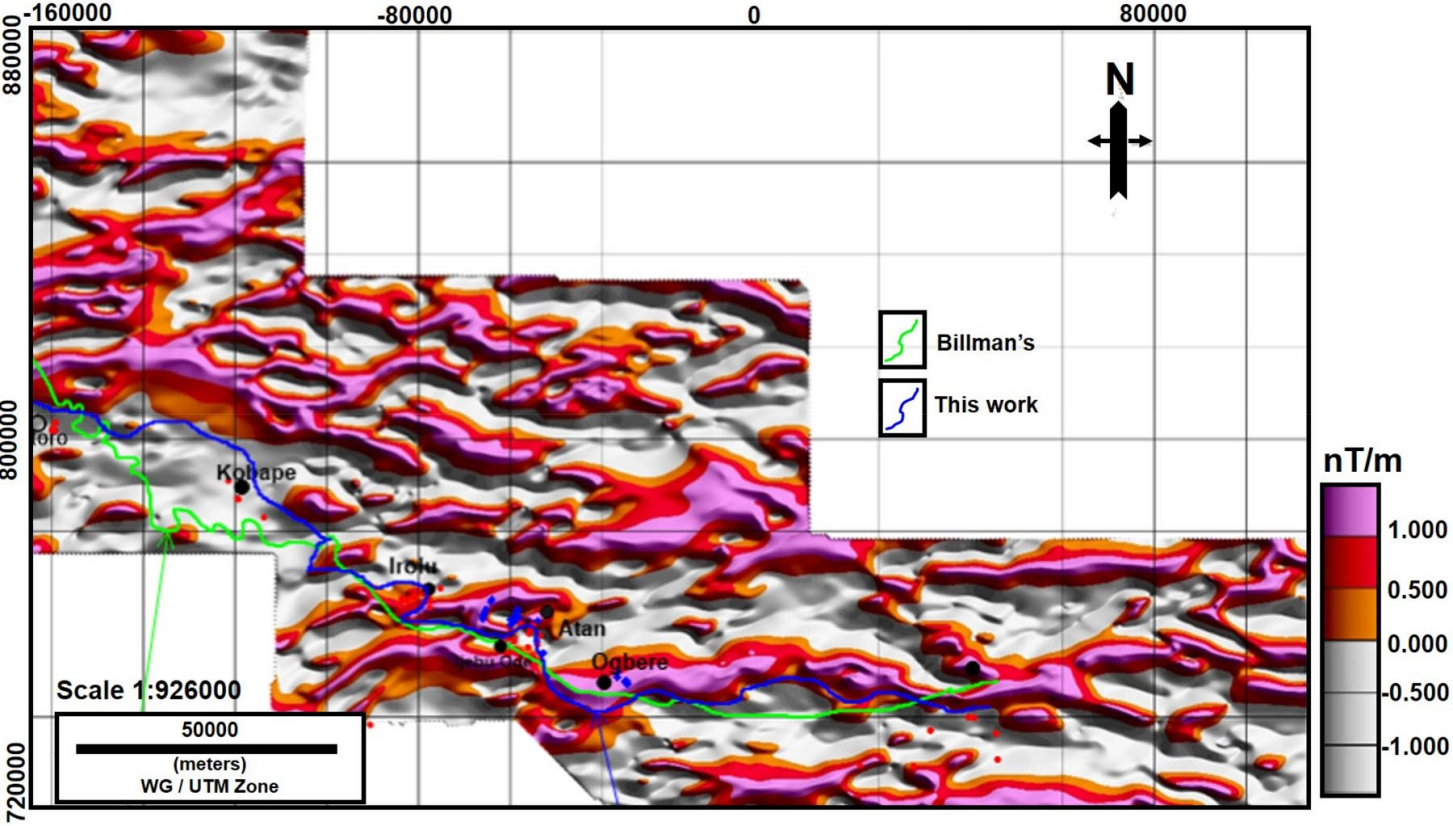
TDR Map with the geologic boundary from^36^ in green and the newly proposed boundary from this work. The red points are inferred sedimentary rocks while the blue points are inferred basement rocks. In addition, the VES points interpreted as basement and sedimentary rocks are overlain on the map. Note: The figure was made using Oasis Montaj software (https://www.seequent.com/products-solutions/geosoft-oasis-montaj/) and edited in CorelDRAW2017, https://www.corel.com/en/.

Furthermore, the geologic boundary and structural lineaments such as faults were enhanced by various derivatives of the magnetic field data such as the TDR of RMI (Fig. 4a) and the TDR of transformed data (Fig. 4b). The TDR of transformed data revealed a very significant trend. Here, the eastern side of the geological map is seen to correlate with contact revealed by the TDR (Fig. 5). Moreover, the TDR transformed data was further enhanced to show the zero values, which were interpreted as the geophysical contact between the basement and sedimentary terrains (Fig. 5). The magnetic lineaments that correspond to faults are generally trending in WSW–ENE and E–W directions while the BS boundary aligns in a NW–SE direction (Fig. 5).

### Vertical electrical sounding

The results of the 104 VES surveys are provided in Appendix I. Importantly, the VES stations show two main terrains that include either sedimentary or crystalline rocks (Figs. 7, 8, 9). The sedimentary rocks are interpreted as part of the Eastern Dahomey Basin while the crystalline rocks are part of basement complex terrain of south western Nigeria. In both terrains, topsoil and laterites are dominant in the upper parts of the succession. The lateritic covers frequently have variable resistivity values from very low e.g., 18.2 Ωm at VES 30 (Oke Ola) to very high values of 7223 Ωm at VES 36 (Ago-Iwoye). Average resistivity value for the lateritic layer in the study area is about 898 Ωm. In terms of stratigraphic positioning, the laterites are commonly beneath the topsoil and represents thin layers with thicknesses of 0.2–1.5 m in the sedimentary terrains (Appendix I). Thickness of the laterites can remarkably reach up to 10–25 m in the basement terrains in localities such as in Ogbere, Oke Eri and Ago Iwoye (Appendix I).

**Figure 6:**
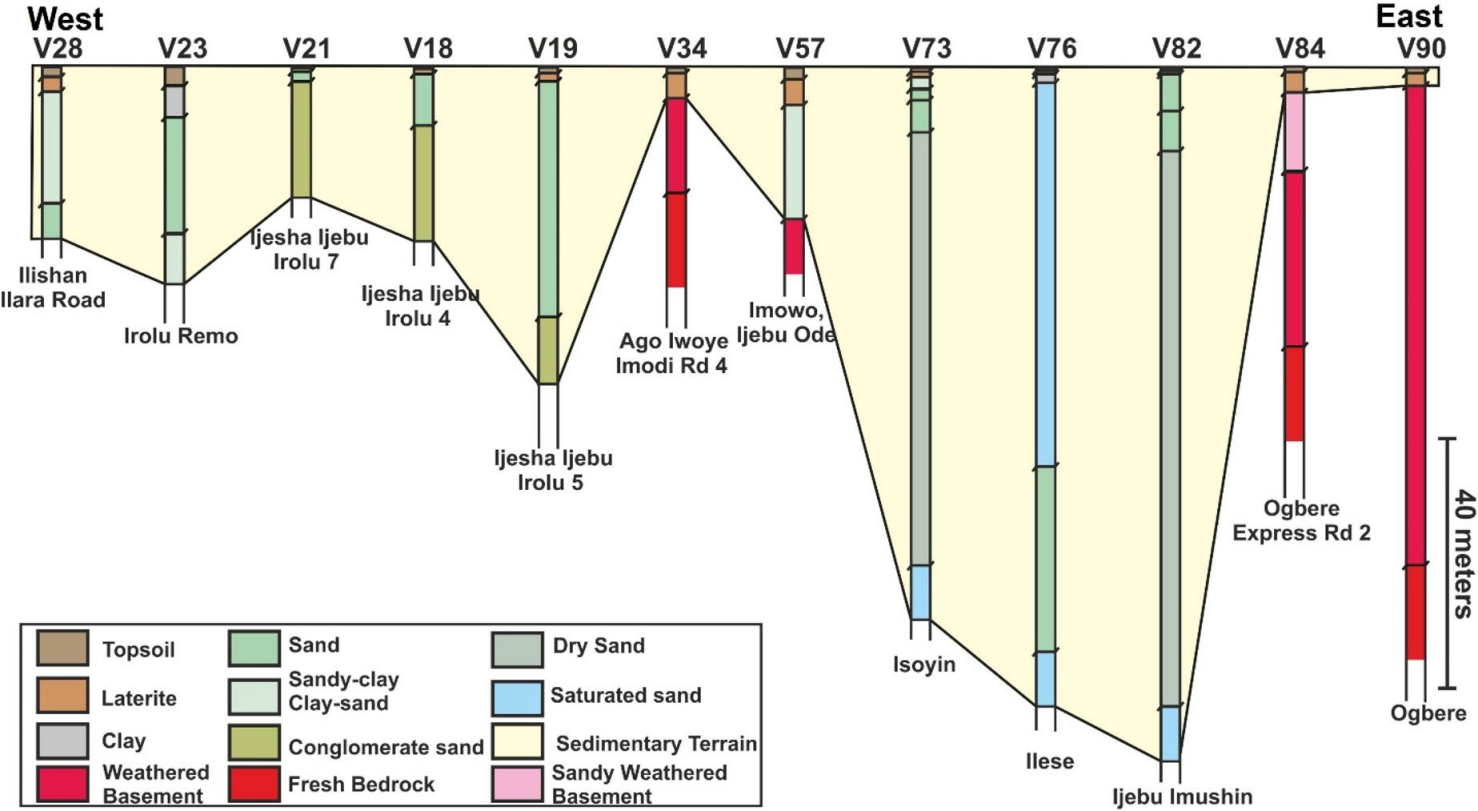
Regional VES profile 1 from Ayetoro through Ijebu Ijesha Irolu to Ogbere showing the different geologic terrains from west to east. The sedimentary rocks are interpreted to onlap the basement complex in the eastern part of the profile. The location of the VES transect is shown in Fig. 1a.

**Figure 7:**
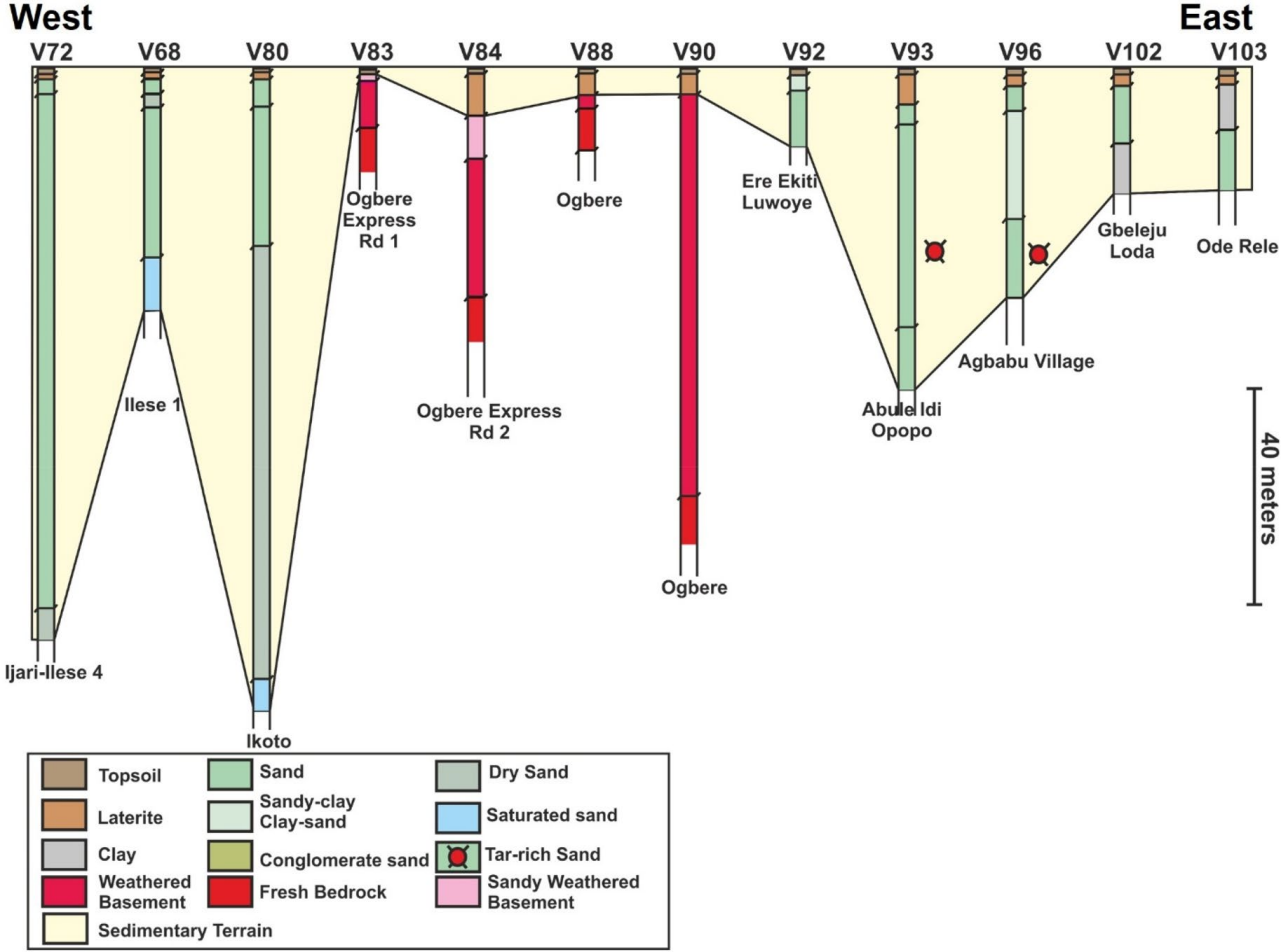
Regional VES profile 2 from Ilishan through Ago-Iwoye to Ogbere showing the transition from sedimentary terrain to basement complex rocks. The location of the VES transect is shown in Fig. 1a.

**Figure 8:**
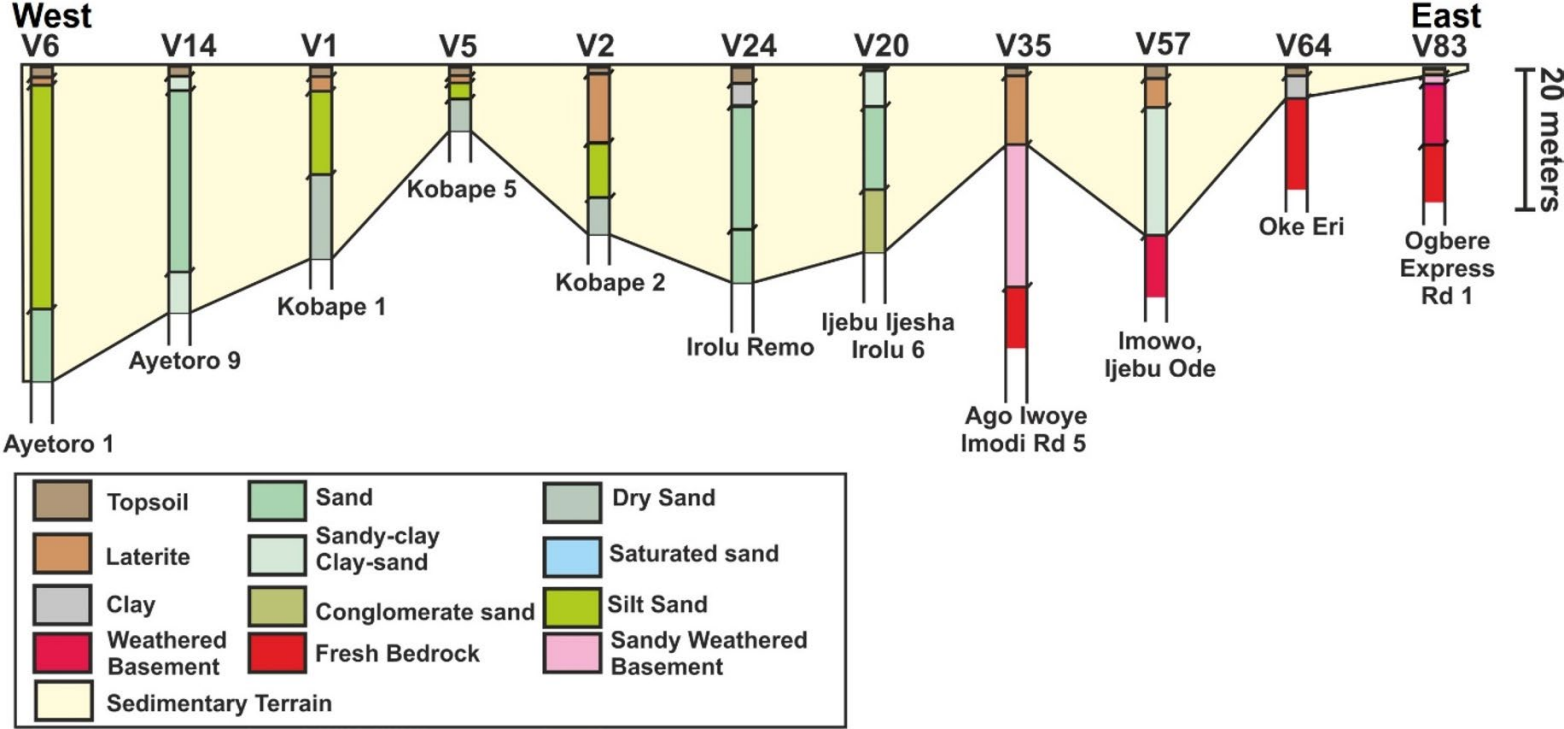
Regional VES profile 3 from Ijari-Ilese through Ogbere to Ode Rele showing how the basement complex is onlapped on the west and the east by sedimentary rock. The location of the VES transect is shown in Fig. 1a.

**Figure 9:**
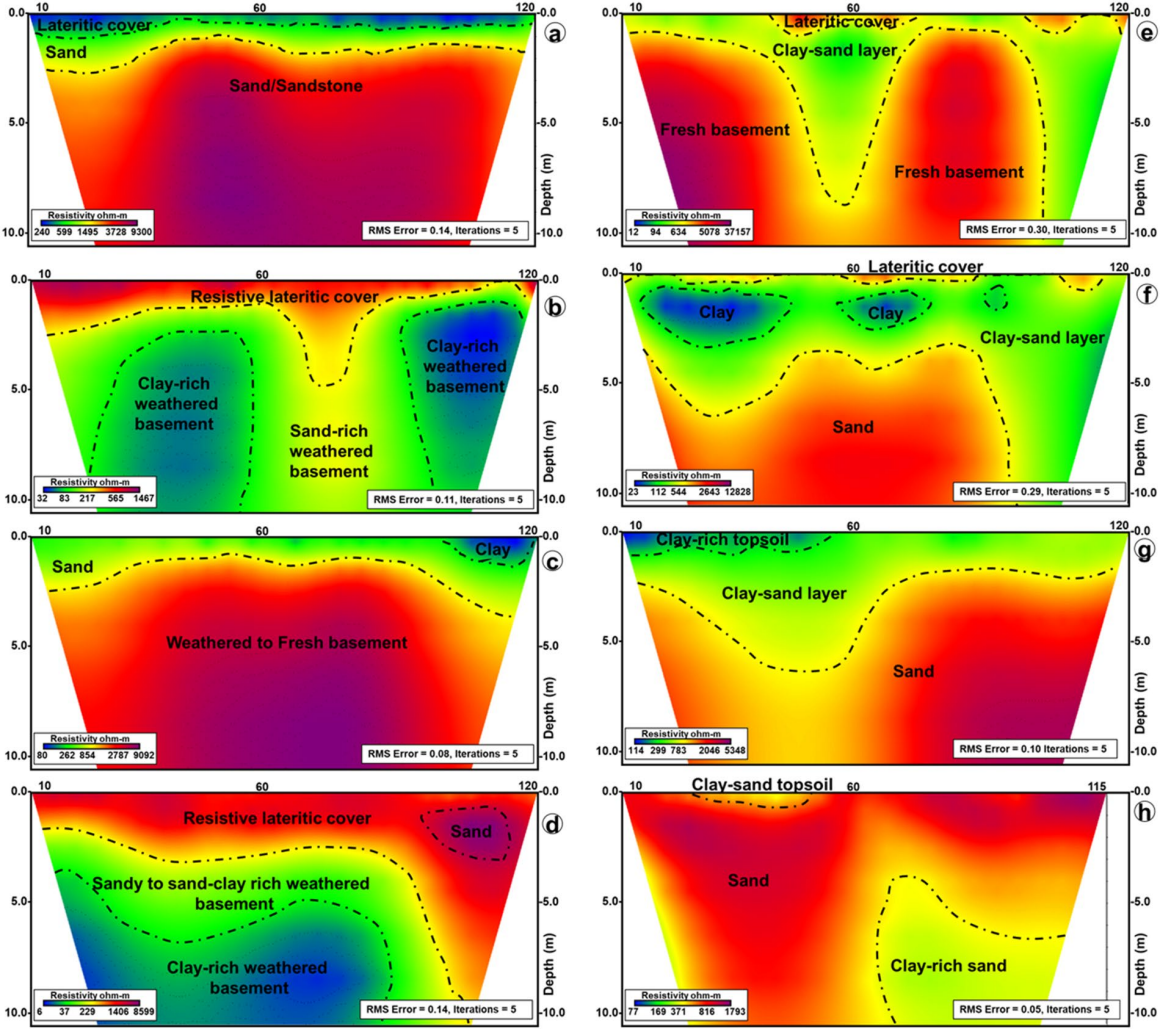
ERT pseudo sections showing the geoelectric layers (stratigraphy) of (**a**) Ago-Iwoye -Ijesha Ijebu 1, (**b**) Ago-Iwoye -Ijesha Ijebu 2, (**c**) Ago Iwoye-Imodi 1 and (**d**) Ago Iwoye-Imodi 2, (**e**) Atan -Erunwon Road 1, (**f**) Atan-Erunwon Road 2, (**g**) Oke Eri-Ogbogbo Road 1 and (**h**) Oke Eri-Ogbogbo Road 2.

Lithologies dominant in the sedimentary terrain includes clay, sand, sandstone, clay-rich sand, conglomeratic sand, and silty sand (Figs. 6, 7, 8). The sands in localities such as Kobape, Ijari, Oke Eri, Ilese and Ijebu Imushin are interpreted as dry or saturated sands. The dry sands have resistivity values that range from 2115 Ωm (VES 4) to 14, 813 Ωm (VES 67) and can occur at varying depths from about 7.7 m up to 65 m in the subsurface (Appendix I, Figs. 6, 7, 8). In addition to the dry sands, saturated sands are also common in the sedimentary terrain with resistivity values of 1494 Ωm to 9446. Saturated sands are common in places such as Isoyin, Ilese, and Ikoto (Appendix I). Clay or clay-dominated layers in the study area have low resistivity values such as 67 Ωm (VES 23 at Irolu Remo) up to 87.4 Ωm (VES 13 at Ayetoro 8) and occur at varying depths. The conglomeratic sands are restricted to the Ijesha Ijebu-Irolu axis, having resistivity that varies between 2512 Ωm (VES 17) to 6189 Ωm (VES 18). Resistivity of the silty sands can vary from 297 Ωm (VES 13) to 1377 Ωm (VES 1). Silty sands were found in Kobape, Ayetoro, Ijari, and Oke Eri (Appendix I, Figs. 7, 8, 9). In addition to the different types of sands, oil or tar bearing sands, which are mainly composed of sands, heavy oil (bitumen) and clays^38^ are interpreted on the Ondo axis in localities such as Abule Idi opopo, Omila, Lamudifa, Agbabu and Kajola (Appendix I). These tar sands have high resistivities of up to 5445 Ωm (e.g., VES 96 at Agbabu).

The main crystalline rocks interpreted from the VES stations are weathered basement, fractured basement and fresh basement/bedrock. Resistivities of these rocks are in the order of 141 Ωm to 1235 Ωm, 1008 Ωm, and 218.4 Ωm to 73,170 Ωm. A fractured basement was only interpreted at Oke Eri-Ogbogbo Rd 4 (VES 44). The weathered basement rocks in the study area are further characterized into clayey weathered basement (resistivities of 28–105 Ωm), partially weathered basement (resistivities of 1289–8789 Ωm), sand-clay rich weathered basement (resistivities of 162–900 Ωm) and sandy weathered basement (resistivities of 148–1268 Ωm). The depth to the basement can vary dramatically from being very shallow (e.g., 2.4 m at Ogbere Express Rd.1, VES 83; Appendix I and Fig. 6) in some places to being very deep in others (e.g., 79 m at Ogbere, VES 90; Appendix I and Fig. 7).

In summary, 9 VES at Ayetoro, 5 VES at Kobape, and all the 16 VES at Ilishan-Ijesha-Ijebu axis indicated sedimentary terrains (Appendix I). Along Ago-Iwoye to Imodi road, the 10 VES results established a basement complex terrain (Figs. 6 and 7). The transition between the basement complex and sedimentary terrain is established at Oke-Eri to Ogbogbo axis Here, 6 of the VES surveys revealed basement complex rocks while the remaining 2 have sedimentary rocks (Appendix I). Similarly, along Erunwon- Atan road, 6 of VES surveys show sedimentary basin rocks while the remaining 1 showed lithology that is characteristic of the basement complex (Appendix I). The 16 VES within Ijari-Ilese-Ijebu Imushin axis and the 13 VES surveys within Okitipupa all pointed to a sedimentary terrain while all the VES at Ago Iwoye-Oke Eri axis and Ogbere indicated rocks of the basement complex (Appendix I, Figs. 6, 7, 8). Additionally, the three VES profiles show distinct stratigraphic or depositional architectures where the sedimentary rocks of the Eastern Dahomey Basin onlap onto the basement rocks in places like Ogbere Express road 1, Ogbere, Ago-Iwoye, Imowo, and Oke Eri (Figs. 6, 7, 8). This onlapping relationship signifies that the sedimentary rocks of the study area are younger than the crystalline rocks.

### Electrical resistivity tomography

The results of the eight (8) ERT are presented as inverted resistivity pseudosections in Fig. 9. All the profiles are dominated by three geoelectric layers related to either sedimentary or crystalline rocks. It is equally important to mention that some of the ERT profiles were shot along some VES lines in order to corroborate the interpreted stratigraphy and subsurface rocks from the VES surveys.

### Profile 1 and 2: Ago-Iwoye to Ijesha-Ijebu Road 1 and 2

This profile is located at Ijesha-Ijebu and was carried out between two sedimentary terrains i.e., VES 15 and VES 20. The upper geoelectric layer is lateritic in nature and with resistivity between 200 and 500 Ωm (Fig. 9a). Underneath this layer is a dry sand layer with resistivities of 600–1000 Ωm, and average thickness of 1.5 m. The last layer interpreted is sandstone with relatively high resistivity values of 1000–9000 Ωm (Fig. 9a). This last layer could also be conglomeratic in nature based on its high resistivity value towards the base of the profile. Profile 2 was shot across VES 35, VES 31 and VES 32 (all defined as belonging to basement terrains, Appendix I). Profile 2 revealed a resistive lateritic topsoil, which extends throughout the entire profile (Fig. 9b). Underlying the lateritic layer is a sand-rich weathered basement with resistivity values of 200–560 Ωm. On the western and eastern side of the profile, the sand-rich weathered basement is flanked by clay-rich weathered basement (Fig. 9b). Here, the basement has resistivity of 30–80 Ωm.

### Profile 3 and 4: Ago-Iwoye-Imodi 1 and 2

The three geoelectric shown by profile 3 are clay, sand and weathered/fresh basement (Fig. 9c). Resistivity of the sand layer varies from 260 to 950 Ωm, and forms of the topsoil layer from the western part to the middle of the profile. On the eastern part of the profile, the layer at the top is a clay-rich unit with resistivity of about 80 Ωm (Fig. 9c). The weathered to fresh basement has resistivity values of 2700–9050 Ωm. Profile 4 also shows three geoelectric layers. This includes a resistive lateritic cover with a thickness of about 2.5 m at the beginning of the profile, which reaches up to 9 m towards the eastern end (Fig. 9c). Resistivity of this layer is about 1500 Ωm (Fig. 9d). The underlying layer is a sandy to sand-clay rich weathered layer with resistivity of 50–230 Ωm. This is in turn underlain by a clay-rich weathered basement with resistivity of less 30 Ωm (Fig. 9d). Thickness of the sand-clay rich weathered layer is about 2–3 m.

### Profile 5 and 6: Atan-Erunwon Road 1 and 2

Profile 5 was made along Atan-Erunwon road close to Odoti Ilugun, a basement complex terrain (Fig. 9e). The topsoil in the area is interpreted to have varied resistivity from being low to high. The low resistive area is the clay-sand cover (about 100 Ωm) while the high resistive area contains the resistive lateritic cover (about 100 Ωm). The most part of the tomography revealed a highly undulating fresh basement rock (resistivity of 6000–35,000 Ωm). Conversely, Profile 6 was made close to a sedimentary terrain and it revealed three main geoelectric layers. These are the topsoil of laterite to sand-clay rich layers, a clay-rich sand layer that often contains nuggets of clay patches, and a basal unit of sand (Fig. 9f).

### Profile 7 and 8: Oke Eri-Ogbogbo Road 1 and 2

Profile 7 is closer to Ogbogbo (sedimentary terrain) than to Oke-Eri (basement terrain) around VES 42 location (Fig. 9g). From the western part of the profile, the topsoil is clay-rich (resistivity of about 110 Ωm) and changes into a clay-sand rich layer (resistivity of about 290–780 Ωm) towards the eastern part of the profile. This latter layer represents the second geoelectric layer on the profile and it has thickness of 2–5 m (Fig. 9g). At the base of Profile 7 is a sand layer with resistivity of more than 2000 Ωm. Similarly, Profile 8 also show three geoelectric layers. A thin clay-sand layer at the beginning of the profile and a second layer that is sand-rich, but also forms part of the topsoil for the remainder of the profile (Fig. 9h). The sand layer can extend to the base of the profile. However, in the eastern part of the profile it is replaced by a lower resistivity (165–370 Ωm) and clay-rich sand (Fig. 9h).

## Discussion

### Defining a new rock boundary using aeromagnetic and electrical resistivity results

In order to combine the results from the processed aeromagnetic data with the geologic terrains inferred from the Electrical Resistivity surveys, the positions of the VES points were plotted on the transformed data in Fig. 5. On this map, the positive values (greater than zero) are considered source of the magnetic gradients, which are basement rocks while the negative values are non-magnetic sedimentary rocks. The zero values represent the boundary between the basement and sedimentary rocks^11^.

**Figure 10:**
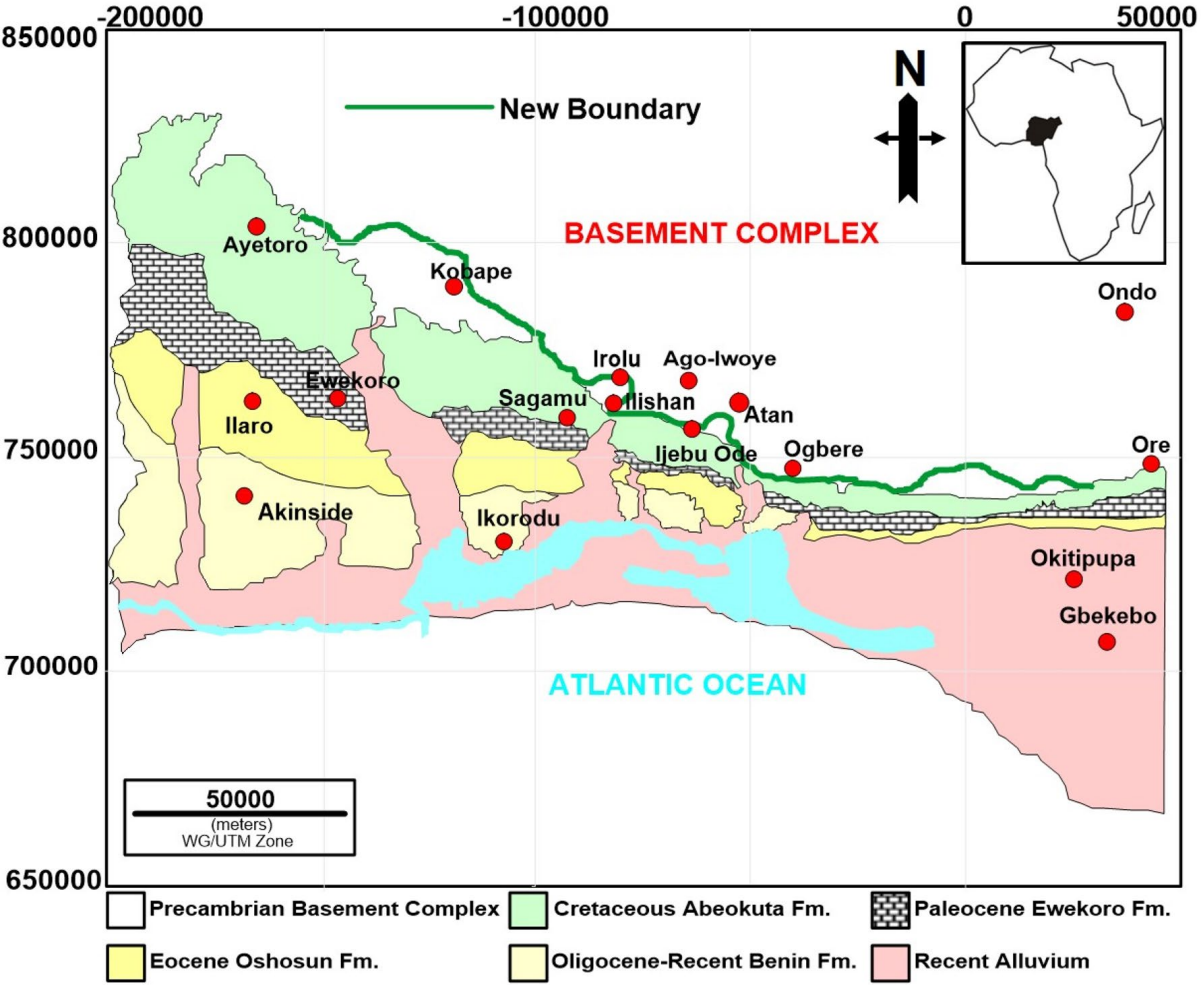
Geological map of the Eastern Dahomey Basin^36^ showing the revised eastern boundary of the basin as defined from aeromagnetic tilt derivative boundary edge, ERT and VES interpretation. Note: The figure was drawn in CorelDRAW2017, https://www.corel.com/en/ while the new boundary was taken from Figs. 3, 4 and 5.

In comparison with the geological map of^25^ (Fig. 1a), this study has refined the boundary between the crystalline rocks of the basement complex of Nigeria and the sedimentary rocks of the Eastern Dahomey Basin (Fig. 10). Localities such as Ago Iwoye, Ogbere and Atan show positive Tilt Derivative values and are regarded as sources of magnetic anomalies. Thus, interpreted as basement complex terrains in a similar way as^27^. More so, at Ago-Iwoye, the geology is not controversial as many outcrops of basement rocks have been previously mapped there^39–41^. The VES points at Ago-Iwoye also correlate to positive values of the Tilt Derivatives suggesting the source of the magnetic gradient and a basement complex terrain (Fig. 5). Ayetoro as interpreted in this work also agrees with the definition of^27^. This is markedly evident from the good match or correlation between the VES and aeromagnetic data interpretation. The Tilt Derivatives values, with a negative tilt value also suggest that Ayetoro is far from the magnetic gradient source and support its classification as a sedimentary terrain (Fig. 5).

On the contrary, Kobape was previously defined as a transition zone between the basement and sedimentary terrains^25,42^. However, the VES surveys here show that it is a sedimentary terrain (Fig. 6). This interpretation is validated by the TDR, which showed negative values signifying a non magnetic environment (Fig. 5). Although, the large area covered by the negative tilt derivative values create doubts as to the coverage of the sedimentary terrain in this area. The negative value could also have been due to the thickness of the basement cover (i.e., the overburden), which has probably reduced the intensity of the magnetic anomaly. Thus, resulting in large distance from the source (negative tilt derivative values).

Irolu and Ijesha-Ijebu areas were previously classified as basement complex terrains by^27^. However, Bayewu et al.^43^ described a non-conformity at Ijesha-Ijebu, where a conglomeratic bed above a weathered layer rock was identified, suggesting the beginning of the sedimentary terrain. Irolu on the other hand, to the west of Ijesha-Ijebu was interpreted as a sedimentary basin in the work (Fig. 7, Appendix I). An indication that the description of these areas as part of the basement complex terrain by^36^ is misleading. From this study, the TDR map uniquely shows Irolu as a transition zone because it largely displayed a gradient value of zero (Fig. 5).

Towards the southeastern part of Ago-Iwoye is Atan, which is also a basement complex terrain. The VES surveys here also indicate a basement terrain. However, on its southwestern toward Ijebu-Ode, the VES surveys show a transition into the sedimentary terrain. These areas defined as sedimentary terrains from the VES were described as part of basement complex by^36^. To resolve this classification, the TDR map in Fig. 8 is prioritized where it shows that these areas are either on the zero or negative value of the tilt derivatives, suggesting either a basement-sedimentary transition or sedimentary terrain.

### Uncertainties in previous geological mapping of the basement-sedimentary terrain (BS) boundary

The boundary between the basement complex and the Eastern Dahomey Basin was first defined by^36^ while describing the offshore stratigraphy and paleontology of the Dahomey embayment. This map like many other geological maps could have been spatially incorrect^44^. The boundary by^36^ might have been solely defined based on geological mapping without emphasis for positioning and correct use of geographical coordinates. Moreover, the Billman’s map could have been done on a regional scale without cognizance for differences in formation types, especially in onshore areas. Since the stratigraphy of the Eastern Dahomey Basin is dramatically different from Onshore to Offshore areas^23^. Hence, it is pertinent to incorporate multiple geologic and geophysical data to properly define the eastern boundary of the Dahomey Basin. Conventional geological mapping should be supported by geophysical data such as regional 2D seismic, borehole data, gravity and electromagnetic data^45–47^. This is in addition to other remotely sensed data^1,2,7^.

### Implications

Using high-resolution aeromagnetic data and subsurface electrical resistivity data, the boundary between the basement complex rocks of southwestern Nigeria and the sedimentary rocks of the Eastern Dahomey Basin has been redefined. The electrical surveys include both Vertical Electrical Soundings and Electrical Resistivity Tomography. These surveys showed the presence of two main geological terrains in the study area, sedimentary and basement complex. Rocks characterized in the sedimentary terrain are clay, sand, sandstone, clay-rich sand, conglomeratic sand, and silty sand while the basement rocks are typified by the weathered basement, fractured basement and fresh basement/bedrock. Significantly, the TDR map of the transformed data offered supporting evidence for the classification of the terrains. Positive values on the TDR map are basement rocks while the negative values are sedimentary rocks. By integrating the interpretation from both geophysical data, the boundary between the Eastern Dahomey Basin and the basement complex of Nigeria is adjusted from the^27^ geological map in the following areas:

- Kobape near Abeokuta has been reclassified to be within the sedimentary basin on the new map.
- Ijesha-Ijebu-Irolu axis has been adjusted as an area within the Eastern Dahomey Basin as against being part of the basement complex rocks of southwestern Nigeria.
- Atan-Ijebu axis was readjusted to include sedimentary rocks as against being within the basement complex terrain.
- Ogbere axis was changed to reveal new observation. Even though the old map delineated part of this axis to be within the basement complex terrain. Our investigation shows that the basement area extends more to the south.

## Data and methods

### Aeromagnetic data

Aeromagnetic data was acquired from the Nigeria Geological Survey Agency (NGSA). The data was part of the nationwide airborne survey carried out by Fugro Airborne Surveys (Fugro) sponsored by the Nigeria Geological Survey Agency (NGSA) from 2003 to 2009 (Fig. 1b). The aeromagnetic data were collected at a flight line spacing of 500 m, terrain distance of 80 m, on a scale of 1: 100,000, and oriented in NW–SE direction with a tie line spacing of 2000 m. The Total Magnetic Intensity (TMI)over the study area are 240-Igangan, 260-Ogun, 261-Ibadan, 280- Lagos, 262-Apomu, 281-Lekki, 282-Okitipupa, and 283-Siluko (Fig. 1c). These grids cover the basement complex-sedimentary basin boundary of the Eastern Dahomey Basin. Oasis Montaj software was used for the processing and interpretation of the data. The regional magnetic field determined by fitting a 2-dimensional first-degree polynomial surface to the total field data using the trend analysis least square^48,49^ method was subtracted from TMI to obtain the residual magnetic Intensity (RMI)^50^. Subsequently, RMI was gridded using kriging algorithm^51–54^. Gridding was done to interpolate the data from the measurement locations to nodes of a regular mesh thereby, creating a fundamentally new different construct of the data^55^. The resultant gridded RMI map (Fig. 3a) was filtered using the fast Fourier transformation. Filters such as, ‘upward continuation’ and ‘reduction to equator’ to enhance deep magnetic sources and correct for the magnetic inclination and declination respectively^50,56^. In a final processing stage, the Tilt Derivative Angles (TDR) (Miller and Singh 1994) was applied for edge detection^57,58^.

### Correction of aeromagnetic data

Correction for inclination and declination of the Earth’s Magnetic field was based on the Reduction to the Equator (RTE) because the study area has relatively low latitudes. Although, reduction to the pole (RTP) as a means of reducing the maps made anywhere except at very low latitudes into what they would be if the inclination of the magnetic field were 90°^59^. However, the RTE is more reliable at high latitudes than RTP at low latitudes^60,61^. Even though the magnetic field is more complex at the equator than the actual magnetic field at the pole, a reduced to the equator map has been described as less complex and more accurate than a reduced to the pole map^62^. For this study, the RTE reduction was done to center the peaks of magnetic anomalies over their sources in order to enhance interpretation of the data whilst still preserving their geophysical meaning. In addition to the RTE corrections, an upward continuation^63,64^ filter was applied to image deeper source of magnetic gradients since the Dahomey Basin is a relatively deep rift basin^65^. The upward continuation filter was used to smooth the data by reducing the high frequencies, remove noise, and the effects of shallow sources. An upward continuation to 2000 m was used to transform the magnetic anomalies as if the observations were made at that plane.

### Boundary and edge detection

Over the years, many methods have been employed to detect causative source edges in potential field maps based on combinations of the horizontal and vertical derivatives data. Such methods include tilt angle (TDR)^11^, the total horizontal derivative of the tilt angle (THDR)^66^, the Theta map^67^, the horizontal tilt angle (TDX)^68^, the wavelet analysis^69^, the normalized horizontal derivative^70^, the tilt angle of the horizontal gradient^71^ and Canny edge detection algorithm^72^. Other recent methods include the tilt angle of the balanced total horizontal derivative (TBHD)^73^, use of logistic function^74^, combination of the analytic signal amplitude and the generalized logistic function^75^ and use of logistic function and the total horizontal gradient^76^.

The TDR, the angle between the total (x and y) horizontal derivatives and the first vertical derivative was used to enhance the edges of magnetic sources. A positive tilt angle corresponds to the source while negative value indicate distance from the source^11^. The zero values which are of interest correspond to the edge or near edge and abrupt changes between positive and negative anomalies. Such abrupt changes are common along faults, which are generally depicted by magnetic lineaments. Even though this method has been found to be sensitive to dip and magnetization effects^77^. However, it is limited in its ability to make both the shallow and deep sources visible simultaneously^11^ and make it suitable for geological boundary mapping^78^.

## Resistivity measurements

Electrical resistivity method that consists of Vertical Electrical Soundings (VES) and Electrical Resistivity Tomography (ERT) techniques were adopted. One hundred and four (104) VES stations and eight (8) ERT surveys were carried out along the basement complex-sedimentary terrain boundary defined by Billman 1976. The VES data were acquired with the Campus Ohmega resistivity meter. Schlumberger electrode was used for the data acquisition because of its convenience for VES survey^79,80^. The field procedure involved the use of four electrodes i.e., 2 currents and 2 potentials. Data were presented as depth sounding curve comprising a plot of apparent resistivity value (y-axis) against the electrode spacing (x-axis) on a log–log graph that is of the same modulus as the interpretation master curve. The interpretation of VES data was both qualitative and quantitative. Qualitative interpretation involved drawing inferences from the plotted curves while quantitative interpretation involves the use of partial curve matching 2-layer master and auxiliary curves. The apparent resistivity plots were iterated on the master and auxiliary curves to get layer resistivities, depths and thicknesses of the VES stations (Appendix I).

## Electrical resistivity tomography (ERT)

ERT combines vertical and horizontal profiles to produce a 2D or 3D image of geo-electric layers in the subsurface^21,29,81^. This study used a data acquisition procedure consisting of many electrodes connected to a multi-core cable^82^. The Wenner electrode configuration^83–85^ was employed here where 5 m electrode spacing, “1a” was used for the first layer, n = 1. The four electrodes take a successful reading at any given point in time and were separated by 5 m each when the first reading was taken. For the first measurement, electrodes number 1, 2, 3 and 4 represented first current electrode C1 or A, first potential electrode P1 or M, second potential electrode P2 or N and second current electrode C2 or B, respectively. After completing the sequence of measurements with “**1a**” spacing, the next sequence, n = 2 measurements with “**2a**” (10 m) electrode spacing was made. The only difference in the measurement at n = 1 and n = 2 is the spacing between the electrodes, which was 5 m and 10 m, respectively. Distance of movement of the electrodes on the survey profile line was 5 m but with electrode spacing of 10 m. In this study, a profile length of 120 m was used throughout the entire survey except for the profile at Oke Eri-Ogbogbo road, which was a profile of 115 m length.

Processing and interpretation were done by plotting the data from a 2-D imaging survey into pseudosections that represent apparent resistivity values in a pictorial form^80^. The processing includes automated forward modeling and data inversion using DIPROfWin software. The inversion routines were based on the smoothness constrained least squares optimization technique and an Active Constraint Balancing, ACB^86–88^. The forward resistivity calculations involved applying a Finite Difference Modeling (FDM) or Finite Element Modeling (FEM) approximations based on an iterative algorithm. FDM methods^89^ are much more flexible with regard to the conductivity and structure. In addition, the amount of numerical work required for the FEM is huge, because the corresponding matrices are not as sparse as those from FDM^90^. Therefore introduced the FDM into the forward modeling as a result of heavy computation burden of the FEM^91^. The FDM component of the software was therefore used to generate theoretical models, which were compared with the observed resistivity data in order to obtain final pseudosections that best signify the geology/stratigraphy of the study area. Furthermore, the ACB method was used to determine the spatially varying Lagrangian multiplier at each of the parameterized blocks of the model during the inversion process to enhance both resolution and stability^92,93^ RMS error and the number of iterations provide constraint on the quality of the final pseudosections. In the study area, the maximum RMS error obtained was 3%, while iteration of 5 was used for all the ERT profiles. This shows that the resistivity sections are of good quality and representative of the subsurface geology.

## Supplementary Information

Supplementary Information.

## Data Availability

Data are available in Supplementary information.
