# Supplementary material for: Redefining the boundary between crystalline and sedimentary rock of Eastern Dahomey Basin
Source: Sci Rep. 2021 Mar 3;11:5016. doi: 10.1038/s41598-021-84687-8 (PMC7930266; doi:10.1038/s41598-021-84687-8)
Supplement: Supplementary file 1 — Supplementary Information. [file 41598_2021_84687_MOESM1_ESM.pdf]

Redefining the boundary between crystalline and sedimentary rock of Eastern Dahomey Basin

[1]Ganiyu O. Mosuro*, [1]Niyi-Ola Adebisi, [1]Stephen O. Ariyo, [2]Kamaldeen O. Omosanya, [1]Olateju O. Bayewu, and [3]Moroof O. Oloruntola.

[1]Department of Earth Sciences, Olabisi Onabanjo University, Ago-Iwoye. Nigeria.
[2]Oasisgeokonsult 7052, Trondheim. Norway.
[3]Department of Geosciences, University of Lagos, Lagos, Nigeria.
*mosuro.ganiyu@oouagoiwoye.edu.ng

Appendix I: Interpretation of the 104 vertical electrical sounding stations.

S/NO	VES stations	No of layers	Resistivity (ohm-m)	Thickness (m)	Depth (m)	Inferred lithology	Terrain
VES1	Kobape 1	1	394	1.1	1.1	Topsoil	Sedimentary
		2	565.1	1.4	2.5	Laterite	
		3	1376.9	12.1	14.6	Silt sand	
		4	3810.0			Dry sand	
VES2	Kobape 2	1	152.1	0.8	0.8	Topsoil	Sedimentary
		2	1277	8.9	9.7	Laterite	
		3	863.5	8.6	18.3	Silt sand	
		4	2318.5			Dry sand	
VES3	Kobape 3	1	199.4	1.5	1.5	Topsoil	Sedimentary
		2	447.1	1.9	3.5	Laterite	
		3	247	5.2	8.6	Clayey sand	
		4	1274.4	10.1	18.7	Silt sand	
		5	1745.4			Dry sand	
VES4	Kobape 4	1	313.7	0.9	0.9	Topsoil	Sedimentary
		2	156.4	2.7	3.7	Sandy clay	
		3	2115			Dry sand	
VES5	Kobape 5	1	568.2	0.9	0.9	Topsoil	Sedimentary
		2	1114.2	0.8	1.7	Laterite	
		3	468.2	4.3	6	Silt-Sand	
		4	2451.8			Dry sand	
VES6	Ayetoro 1	1	726.8	1	1	Topsoil	Sedimentary
		2	1651.2	0.9	1.9	Laterite	
		3	410.6	32.5	34.4	Silty Sand	
		4	963.6			Sand	
VES7	Ayetoro 2	1	58.8	0.5	0.5	Topsoil	Sedimentary
		2	583	4.9	5.3	Laterite	
		3	137.9	36.2	41.5	Sandy Clay	
		4	905.3			Sand	
VES8	Ayetoro 3	1	166.5	0.8	0.8	Topsoil	Sedimentary
		2	471.7	6	6.7	Laterite	
		3	347.1	15.3	22.1	Silty Sand	
		4	296.9			Clayey Sand	
VES9	Ayetoro 4	1	456.7	0.9	0.9	Topsoil	Sedimentary
		2	1007.3	3	3.9	Laterite	
		3	191.7	27.1	31	Clayey Sand	
		4	2464			Sand	
VES10	Ayetoro 5	1	321.5	1.1	1.1	Topsoil	Basement
		2	1177.8	4.4	5.5	Laterite	

S/NO	VES stations	No of layers	Resistivity (ohm-m)	Thickness (m)	Depth (m)	Inferred lithology	Terrain
		3	134.5	15.8	21.4	Sandy clay	
		4	8369.9			sandstone	
VES11	Ayetoro 6	1	2685.4	0.9	0.9	Topsoil	Sedimentary
		2	511.3	8.4	9.3	laterite	
		3	1052.7	2.8	12.1	Sand	
		4	295.4			Clayey sand	
VES12	Ayetoro 7	1	73	1.1	1.1	Topsoil	Sedimentary
		2	150.6	3.1	4.2	Sandy Clay	
		3	294.7	29.2	33.4	Sand	
		4	137.2			Sandy Clay	
VES13	Ayetoro 8	1	31.1	1.2	1.2	Topsoil	Sedimentary
		2	137.4	2.8	4	Sandy clay	
		3	297.4	28.6	32.6	Silty Sand	
		4	87.4	34.4	67	clay	
		5	1263.4			Sand	
VES14	Ayetoro 9	1	48.3	1.2	1.2	Topsoil	Sedimentary
		2	126.5	1.6	2.8	Sandy Clay	
		3	419.1	21.3	24.2	Sand	
		4	179.3			Sandy Clay	
VES15	Ijesha Ijebu - Irolu 1	1	112.4	0.4	0.4	Topsoil	Sedimentary
		2	611	3	3.3	Laterite	
		3	3289			Sandstone/ Conglomeratic	
VES16	Ijesha Ijebu - Irolu2	1	126.8	1.3	1.3	Topsoil	Sedimentary
		2	397.9	7.6	8.9	Laterite	
		3	753.3	49.2	58.1	Sand	
		4	5074.9			Sandstone/ Conglomeratic	
VES17	Ijesha Ijebu - Irolu3	1	65.2	0.4	0.4	Topsoil	Sedimentary
		2	250.1	2.3	2.7	Clayey Sand	
		3	2517.7			Sandstone/ Conglomeratic	
VES18	Ijesha Ijebu - Irolu4	1	247.2	0.9	0.9	Topsoil	Sedimentary
		2	978.7	10.2	11.2	Sand	
		3	6188.6			Sandstone/ Conglomeratic	
VES19	Ijesha Ijebu - Irolu5	1	130.4	1.3	1.3	Topsoil	Sedimentary
		2	549	0.7	2.1	Laterite	
		3	1629.6	37.5	39.6	Sand	
		4	5500.4			Sandstone/ Conglomeratic	
VES20	Ijesha Ijebu - Irolu6	1	74.1	0.1	0.1	Topsoil	Sedimentary
		2	138.8	5.1	5.2	Sandy Clay	
		3	1188.5	11.6	16.8	Sand	
		4	1630.9			Sandstone/ Conglomeratic	

S/NO	VES stations	No of layers	Resistivity (ohm-m)	Thickness (m)	Depth (m)	Inferred lithology	Terrain
VES21	Ijesha Ijebu - Irolu7	1	137.4	0.4	0.4	Topsoil	Sedimentary
		2	568.2	4.3	4.8	Sand	
		3	2942.1			Sandstone/ Conglomeratic	
VES22	Ijesha Ijebu - Irolu8	1	97.4	0.2	0.2	Topsoil	Sedimentary
		2	577.4	4.2	4.4	sand	
		3	2639.7			sandstone	
VES23	Irolu Remo	1	89.5	3	3	Topsoil	Sedimentary
		2	68.6	6.6	9.6	Clay	
		3	412.6	18.6	28.2	Sand	
		4	337.1			Clayey Sand	
VES24	Irolu Remo	1	82.5	1.7	1.7	Topsoil	Sedimentary
		2	34.2	3.3	5	Clay	
		3	230.1	18.2	23.2	Sand	
		4	749			Sandstone	
VES25	Ago Iwoye-Ilishan road by third bridge	1	68.4	1.3	1.3	Topsoil	Transition zone/ Sedimentary
		2	94.4	4.1	5.4	Clayey layer	
		3	95.3	7.6	13	Clayey layer	
		4	601	31.2	44.2	Sand	
		5	800			Weathered Bedrock	
VES26	Ilishan- Irolu	1	82.4	1.9	1.9	Topsoil	Sedimentary
		2	70.8	3.8	5.7	Clay	
		3	182.8	18	23.7	Sandy clay	
		4	212	39.2	62.9	Clayey Sand	
		5	238.3			Sand	
VES27	Ilishan	1	137.3	2	2	Topsoil	Sedimentary
		2	157.4	3.9	5.9	Laterite	
		3	129.6	13.9	19.7	Sandy clay	
		4	196.6			Clayey Sand	
VES28	Ilishan-Ilara Road	1	35	1.1	1.1	Topsoil	Sedimentary
		2	108.3	3.1	4.2	Laterite	
		3	193.8	17.8	22	Sandy clay	
		4	793.4			Sand	
VES29	Ilishan-Ilara Road	1	87.1	2.1	2.1	Topsoil	Sedimentary
		2	22.5	3.8	5.9	Clay	
		3	151.9	12.2	18	Sandy clay	
		4	193.9	20.1	38.1	clayey clay	
		5	411.8			Sand	
VES30	Oke Ola (Ilishan)	1	58.6	1.8	1.8	Topsoil	Sedimentary
		2	18.2	3.1	4.9	laterite	
		3	83.4	7.6	12.5	Clay	
		4	101.3	30.6	43.1	Sandy clay	
		5	192.3			Clayey Sand	
VES31	Ago Iwoye-Imodi Rd 1	1	135.1	0.2	0.2	Topsoil	Basement

S/NO	VES stations	No of layers	Resistivity (ohm-m)	Thickness (m)	Depth (m)	Inferred lithology	Terrain
		2	694.5	7.6	7.8	Laterite	
		3	35.3	8.2	16	Clay	
		4	218.4			Fresh basement	
VES32	Ago Iwoye-Imodi Rd 2	1	56.2	0.4	0.4	Topsoil	Basement
		2	1114.5	3.3	3.7	Laterite	
		3	150.2	10	13.7	Weathered Basement	
		4	815.6			Fresh bedrock	
VES33	Ago Iwoye-Imodi Rd 3	1	410	1.3	1.3	Topsoil	Basement
		2	41.1	4.9	6.2	Clay	
		3	1274.2			Fresh bedrock	
VES34	Ago Iwoye-Imodi Rd 4	1	1763.6	1.1	1.1	Topsoil	Basement
		2	1277.7	5.8	6.8	Laterite	
		3	114.1	12.2	19.1	Weathered basement	
		4	5542.3			Fresh bedrock	
VES35	Ago Iwoye-Imodi Rd 5	1	506.3	0.8	0.8	Topsoil	Basement
		2	7223.8	10	10.8	Laterite	
		3	728.8	21	31.8	Sandy weathered basement	
		4	17559.1			Fresh bedrock	
VES36	Ago Iwoye-Imodi Rd 6	1	590.7	1.1	1.1	Topsoil	Basement
		2	900.2	9.1	10.2	Sandy weathered basement	
		3	6887.2			Fresh bedrock	
VES37	Ago Iwoye-Imodi Rd 7	1	91.5	1.1	1.1	Topsoil	
		2	393.5	10.6	11.7	Weathered basement	Basement
		3	25960.3			Fresh bedrock	
VES38	Ago Iwoye-Imodi Rd 8	1	116.4	1.1	1.1	Topsoil	Basement
		2	120.7	9.2	10.2	Sandy clay	
		3	9819.1			Fresh bedrock	
VES39	Ago Iwoye-Imodi Rd 9	1	70.8	1.4	1.4	Topsoil	Basement
		2	112.3	7.2	8.7	Sandy clay	
		3	2479.2			Fresh bedrock	
VES40	Ago Iwoye-Imodi Rd 10	1	44.2	1	1	Topsoil	Basement
		2	13416.1			Fresh bedrock	
VES41	Oke Eri-Ogbogbo Rd 1	1	38.3	0.8	0.8	Topsoil	Sedimentary
		2	365.7	13.8	14.7	Laterite	
		3	3051.8			Dry Sand	
VES42	Oke Eri-Ogbogbo Rd 2	1	206	1	1	Topsoil	Sedimentary
		2	1268.9	9.4	10.4	Sand	
		3	648.8	20.3	30.7	Silty sand	
		4	2539.3			Dry Sand	

S/NO	VES stations	No of layers	Resistivity (ohm-m)	Thickness (m)	Depth (m)	Inferred lithology	Terrain
VES43	Oke Eri-Ogbogbo Rd 3	1	1010.3	1	1	Topsoil	Basement
		2	2025.9	2.9	3.9	Laterite	
		3	1234.9	24.4	28.2	Sandy Weathered Basement	
		4	11080.3			Fresh Basement	
VES44	Oke Eri-Ogbogbo Rd 4	1	352.8	0.9	0.9	Topsoil	Basement
		2	658.4	5.2	6.1	Laterite	
		3	362.6	10.9	17	Sandy Weathered Basement	
		4	1008.3			Fractured Basement	
VES45	Oke Eri-Ogbogbo Rd 5	1	282.7	1	1	Topsoil	Basement
		2	117.7	10.4	11.3	laterite	
		3	484.8	16.9	25	Sandy Weathered Basement	
		4	1373.3			Fresh Basement	
VES46	Oke Eri-Ogbogbo Rd 6	1	130.9	1.9	1.9	Topsoil	Basement
		2	228.3	12.5	14.3	Laterite	
		3	576			Sandy Weathered Basement	
VES47	Oke Eri-Ogbogbo Rd 7	1	77.6	0.6	0.6	Topsoil	Basement
		2	226.7	4.5	5.1	Laterite	
		3	109.8	11.7	16.8	Sandy Clay	
		4	410.9			Sandy Weathered Basement	
VES48	Oke Eri-Ogbogbo Rd 8	1	410.9	1.5	1.5	Topsoil	Basement
		2	503.5	13.3	14.9	Sandy Weathered Basement	
		3	11130.4			Fresh Basement	
VES49	Erunwon-Atan Rd 1	1	325.5	0.8	0.8	Topsoil	Sedimentary
		2	160.3	2.2	3	Clayey Sand	
		3	858.9	35.6	38.6	Sand	
		4	15552.9			Resistive sandstone	
VES50	Erunwon-Atan Rd 2	1	375.5	0.9	0.9	Topsoil	Basement
		2	828.8	2.3	3.3	Lateritic Clay	
		3	239.9	27.4	30.7	Weathered Basement	
		4	3518.5			Fresh Basement	
VES51	Erunwon-Atan Rd 3	1	273.9	1.7	1.7	Topsoil	Sedimentary
		2	1581.6	20	21.7	laterite	
		3	610.5			Sand	
VES52	Erunwon-Atan Rd 4	1	140.9	0.8	0.8	Topsoil	Sedimentary
		2	825.3	12.7	13.5	Sand	
		3	3907.2			Sandstone	

S/NO	VES stations	No of layers	Resistivity (ohm-m)	Thickness (m)	Depth (m)	Inferred lithology	Terrain
VES53	Erunwon-Atan Rd 5	1	156.3	1.1	1.1	Topsoil	Sedimentary
		2	73.4	2.7	3.7	Clay	
		3	1055.3			Sand	
VES54	Erunwon-Atan Rd 6	1	87.8	0.9	0.9	Topsoil	Sedimentary
		2	1260.9	6	6.9	Laterite	
		3	4768.4			Sandstone	
VES55	Erunwon-Atan Rd 7	1	48.8	0.6	0.6	Topsoil	Sedimentary
		2	740.9	6.5	7.1	Laterite	
		3	7787.7			Sandstone	
VES56	Imowo, Ijebu Ode	1	58.3	1.1	1.1	Topsoil	Basement
		2	304.2	4.5	5.5	laterite	
		3	1267.7	20.2	25.7	Sandy weathered basement	
		4	4611.1			Fresh basement e	
VES57	Imowo, Ijebu Ode	1	323.0	1.7	1.7	Topsoil	Basement
		2	2067.5	5.3	7.1	Laterite	
		3	1172.7	17.5	24.6	Sandy layer	
		4	800.9			Weathered Basement	
VES58	Imowo, Ijebu Ode	3	127.5	0.9	0.9	Topsoil	Basement
			728.0	6	6.9	Sandy Weathered basement	
			2084.6			Fresh Basement	
VES59	Ago Iwoye-Oke Eri 4	3	52.2	1.2	1.2	Topsoil	Basement
			213.7	5.9	7.1	Sandy Weathered basement	
			1580.4			Fresh Basement	
VES60	Ago Iwoye-Oke Eri 5	3	115	1.1	1.1	Topsoil	Basement
			208.9	12.8	13.9	Sandy Weathered Basement	
			87.9			Clayey Weathered Basement	
VES61	Oke Eri Junction 6	4	106.9	0.9	0.9	Topsoil	Basement
			27.5	3.2	4.1	Clayey Weathered Basement	
			161.6	20.2	24.3	Sand Clayey Weathered Basement	
			2984			Fresh Basement	
VES62	Oke Eri	4	231.2	1.3	1.3	Topsoil	Basement
			147.6	2.4	3.8	Sandy Weathered Basement	
			81.3	6.9	10.7	Clayey Weathered Basement	
			1264			Fresh Basement	

S/NO	VES stations	No of layers	Resistivity (ohm-m)	Thickness (m)	Depth (m)	Inferred lithology	Terrain
VES63	Oke Eri	3	171.9	1.3	1.3	Topsoil	Basement
			62.8	15.1	16.4	Clay	
			1185.2			Fresh Basement	
VES64	Oke Eri	3	95.6	1	1	Topsoil	Basement
			30.8	3.5	4.5	Clay	
			4548.3			Fresh Basement	
VES65	Oke Eri	4	1010.3	1	1	Topsoil	Basement
			2025.9	2.9	3.9	Laterite	
			1234.9	24.4	28.2	Weathered Basement	
			11080.3			Fresh Basement	
VES66	Oke Eri	4	331.4	0.7	0.7	Topsoil	Basement
			551.4	3.7	4.4	Laterite	
			253.3	15.9	20.3	Sandy Weathered Basement	
			7599.9			Fresh Basement	
VES67	Ijari-Ilese	1	184.7	0.9	0.9	Topsoil	Sedimentary
		2	114.8	1.5	2.5	Laterite	
		3	734.4	1.5	4.1	Clayey sand	
		4	5011	1.5	5.5	Sand	
		5	14811.8	45.4	51.1	Dry sand	
		6	3691.5			Saturated sand	
VES68	Ilese1	1	193.1	0.8	0.8	Topsoil	Sedimentary
		2	1399.7	1	1.9	Laterite	
		3	4965.4	2.4	4.2	Sand	
		4	9543.1	3.5	7.7	Dry Sand	
		5	11392.9	28.4	36.1	sandstone	
		6	2862.5			Saturated sand	
VES69	Ijari –Ilese1	1	429.8	0.8	0.8	Topsoil	Sedimentary
		2	3833.9	0.7	1.5	Laterite	
		3	1684.8	6.2	7.7	Sand	
		4	2810.7	27.2	34.9	Sand	
		5	7035.9	30.4	65.3	Dry sand	
		6	18037.6			Sandstone	
VES70	Ijari-Ilese2	1	180.5	1	1	Topsoil	Sedimentary
		2	741.7	1.3	2.3	Laterite	
		3	1572.6	5.2	7.5	Sand	
		4	2356.4	22.3	29.8	Dry sand	
		5	18335.1	36.7	66.4	Sandstone	
		6	6533.1			Saturated sand	
VES71	Ijari-Ilese3	1	316.8	0.3	0.3	Topsoil	Sedimentary
		2	63.2	10	10.2	Clay	
		3	3581.9	3.5	13.7	Sand	
		4	10168.3	1.3	15	Dry sand	
		5	13288.7	17.1	32.1	Dry sand	
		6	947.3			Saturated sand	
VES72	Ijari-Ilese4	1	182.5	1.1	1.1	Topsoil	Sedimentary
		2	323.3	0.5	1.6	Laterite	
		3	419.1	3	4.7	Silty Sand	
		4	100000	95.5	100.1	Highly resistive sand	
		5	28276.3			Dry sand	
VES73	Isoyin	1	577.2	0.7	0.7	Topsoil	Sedimentary
		2	704.9	1.3	1.9	Laterite	

S/NO	VES stations	No of layers	Resistivity (ohm-m)	Thickness (m)	Depth (m)	Inferred lithology	Terrain
		3	1271	1.7	3.7	Clayey sand	
		4	1329.1	9	12.6	Sand	
		5	2329.3	6.7	19.3	Sand	
		6	11294.8	61.2	80.5	Dry sand	
		7	9445.5			Saturated sand	
VES74	Isoyin	1	163.9	0.6	0.6	Topsoil	Sedimentary
		2	940.6	0.2	0.8	Laterite	
		3	803.1	16.6	17.4	Sand	
		4	7242.4			Sandstone	
VES75	Ilese	1	533.8	1.1	1.1	Topsoil	Sedimentary
		2	1839.3	2.9	4	Laterite	
		3	4358.6	12.2	16.1	Sand	
		4	16025.4	14.7	30.9	Dry sand	
		5	2971	34.4	65.3	Saturated sand	
		6	1493.6			Saturated sand	
VES76	Ilese	1	85.3	0.5	0.5	Topsoil	Sedimentary
		2	73.6	0.2	0.7	Laterite	
		3	32.5	1.7	2.4	Clay	
		4	2546.4	61.9	64.3	Saturated Sand	
		5	7750.4	33.1	97.4	Sandstone	
		6	4191.7			Saturated sand	
VES77	Ilese	1	182.7	0.5	0.5	Topsoil	Sedimentary
		2	607.4	1	1.5	Laterite	
		3	397.5	7.8	9.3	Clayey sand	
		4	1176.3	17.6	26.9	Sand	
		5	3297.2	98.2	125.1	Sandstone	
		6	923.6			Saturated Sand	
VES78	Ilese	1	804.4	0.5	0.5	Topsoil	Sedimentary
		2	532	1.4	1.9	Laterite	
		3	296	10.2	12.1	Clayey sand	
		4	1233.9	1	13.1	Sand	
		5	518.9	41.4	54.5	Clayey sand	
		6	1084.6	54.7	109.2	Sand	
		7	2540.2			Sand	
VES79	Ikoto	1	500	0.5	0.5	Topsoil	Sedimentary
		2	1331.5	0.6	1.1	Laterite	
		3	465	6.1	7.2	Clayey sand	
		4	4029.5	7.7	14.9	Sand	
		5	22307.3	75.3	90.3	Dry sand	
		6	8808.9			Saturated sand	Sedimentary
VES80	Ikoto	1	686.6	1	1	Topsoil	
		2	421.2	1.1	2.1	Laterite	
		3	237.7	4.8	6.9	Clayey sand	
		4	528.1	26.2	33.1	Sand	
		5	20392.6	82.7	115.8	Dry sand	
		6	2986.4			Saturated sand	
VES81	Ijebu Imushin	1	584.5	0.6	1	Topsoil	Sedimentary
		2	387	4.5	2.1	Clayey sand	
		3	2757.8	0.6	6.9	Sand	
		4	2808.4	2.9	33.1	Sand	
		5	6503.8	37.1	115.8	Dry sand	
		6	3355.1			Saturated sand	

S/NO	VES stations	No of layers	Resistivity (ohm-m)	Thickness (m)	Depth (m)	Inferred lithology	Terrain
VES82	Ijebu Imushin	1	810.7	0.3	0.3	Topsoil	Sedimentary
		2	1288.3	0.6	0.8	Laterite	
		3	2341.9	5.6	6.4	Sand	
		4	12079.6	7	13.4	Sandstone	
		5	14481.3	81	94.5	Dry sand	
		6	6471.3			Saturated sand	
VES83	Ogbere Express Rd.1	1	113.8	1.2	1.2	Topsoil	Basement
		2	649.8	1.2	2.4	Sandy Weathered basement	
		3	8789.2	7.8	10.2	Partially weathered basement	
		4	71661.8			Fresh basement	
VES84	Ogbere Express Rd.2	1	139.3	1.0	1.0	Topsoil	Basement
		2	312.9	8.3	9.3	Laterite	
		3	243.3	6.9	16.2	Sandy Weathered basement	
		4	1289.2	26.3	42.5	Partially weathered basement	
		5	12361.8			Fresh basement	
VES85	Ogbere	1	137.7	0.9	0.9	Topsoil	Basement
		2	633.9	4.4	5.3	laterite	
		3	105.4	6.2	11.5	Clayey weathered basement	
		4	1517.7	18.3	29.8	Partially weathered basement	
		5	9548.8			Fresh basement	
VES86	Ogbere	1	140.3	0.8	0.8	Topsoil	Basement
		2	211.6	4.1	4.9	Laterite	
		3	162.5	6.3	11.2	Sandy clay layer	
		4	945.8	38.5	49.7	Sandy weathered basement	
		5	6290			Fresh basement	
VES87	Ogbere	1	131.1	1.1	1.1	Topsoil	Basement
		2	1557.7	14.7	15.8	Laterite	
		3	931.5	6.4	22.2	Weathered Basement	
		4	8053.5	10.7	32.9	Partially weathered Basement	
		5	73170.4			Fresh Basement	
VES88	Ogbere	1	36.9	1.3	1.3	Topsoil	Basement
		2	110.9	3.9	5.1	Laterite	
		3	104.4	1.3	6.5	Clayey weathered Basement	
		4	1003.5			Fresh Basement	
VES89	Ogbere	1	97.2	0.7	0.7	Topsoil	Basement
		2	1204	25.4	26.1	Laterite	
		3	2590.8	5.2	31.2	Partially Weathered Basement	
		4	5291.7			Fresh Basement	
VES90	Ogbere	1	103	1.1	1.1	Topsoil	Basement
		2	604.6	3.1	4.2	Laterite	

S/NO	VES stations	No of layers	Resistivity (ohm-m)	Thickness (m)	Depth (m)	Inferred lithology	Terrain
		3	3673.3	74.9	79.1	Partially Weathered Basement	
		4	10818.7			Fresh Basement	
VES91	Ogbere	1	41.2	0.5	0.5	Topsoil	Basement
		2	508.8	2.5	3.0	Laterite	
		3	243.9	1.4	4.4	Sandy clay Weathered Basement	
		4	5743.9			Fresh Basement	
VES92	Ere Ekiti Luwoye	1	30	1.3	1.3	Topsoil	Sedimentary
		2	164.4	2.8	4.1	Sandy clay	
		3	759.9			Sand	
VES93	Abule Idi Opopo	1	508.1	1	1	Topsoil	Sedimentary
		2	1315	5.4	6.4	Laterite	
		3	670.7	3.3	9.8	Sand	
		4	2046.3	38.1	47.8	Tar Sand	
		5	1547.9			Sand	
VES94	Ondo	1	196.7	1.1	1.1	Topsoil	Sedimentary
		2	513.6	1.5	2.7	Laterite	
		3	1405.9	12.9	15.5	Sand	
		4	170.4			Sandy clay	
VES95	Omila Village	1	389.2	0.6	0.6	Topsoil	Sedimentary
		2	1221.9	4.3	4.9	Laterite	
		3	625.9	16	20.9	sand	
		4	4892.9			Tar Sand	
VES96	Agbabu Village	1	405.2	0.9	0.9	Topsoil	Sedimentary
		2	338.3	2	2.9	Laterite	
		3	866.2	4.9	7.7	Sand	
		4	284.9	19.9	27.7	Sandy clay	
		5	5445.9			Tar Sand	
VES97	Ore-Ekiti Pupa Express	1	111.7	0.9	0.9	Topsoil	Sedimentary
		2	374.2	6.9	7.9	Laterite	
		3	116.6	16.1	23.9	Sandy clay	
		4	1155.2			Sand	
VES98	Oja Baale	1	114.3	1.7	1.7	Topsoil	Sedimentary
		2	54.3	6.3	8	Clay	
		3	314.6	5.6	13.6	Sand	
		4	949			Sand	
VES99	Lamudifa Ondo	1	110.4	1.1	1.1	Topsoil	Sedimentary
		2	217.9	0.9	2	Laterite	
		3	106.9	3.9	5.9	Sandy clay	
		4	205.6	10.4	16.3	Clayey Sand	
		5	2065			Tar Sand	
VES100	Lamudifa	1	300.4	1	1	Topsoil	Sedimentary
		2	351.9	4.6	5.6	Sand	
		3	5048.6			Dry /Tar Sand	
VES101	Kajola Oju Irin	1	251.3	1.3	1.3	Topsoil	Sedimentary

S/NO	VES stations	No of layers	Resistivity (ohm-m)	Thickness (m)	Depth (m)	Inferred lithology	Terrain
		2	3124.1	5.2	6.5	Resistive lateritic Layer	
		3	5363.5			Tar Sand	
VES102	Gbeleju Loda	1	245	1	1	Topsoil	Sedimentary
		2	270.8	2.2	3.2	Laterite	
		3	769.3	10.3	13.6	Sand	
		4	91.5			Clay	
VES103	Ode Rele	1	59.6	1	1	Topsoil	Sedimentary
		2	404	1	2.1	Laterite	
		3	101	9.3	11.4	Clay	
		4	776			Sand	
VES104	Okiti Pupa	1	574.8	1	1	Topsoil	Sedimentary
		2	530.7	2.4	3.4	Lateritic Layer	
		3	1292.4	12	15.4	Sand	
		4	170. 2			Sandy Clay	
